# Supplementary material for: Shared Treg dysregulation underlies psoriasis vulgaris and atopic dermatitis through the PHF19-PRC2 epigenetic axis
Source: Front Immunol. 2026 Aug 20;17:1932788. doi: 10.3389/fimmu.2026.1932788 (PMC13538097; doi:10.3389/fimmu.2026.1932788)
Supplement: Supplementary file 3 [file DataSheet3.zip › Additional files/Additional file 6/37.CKAP2.forest.pdf]

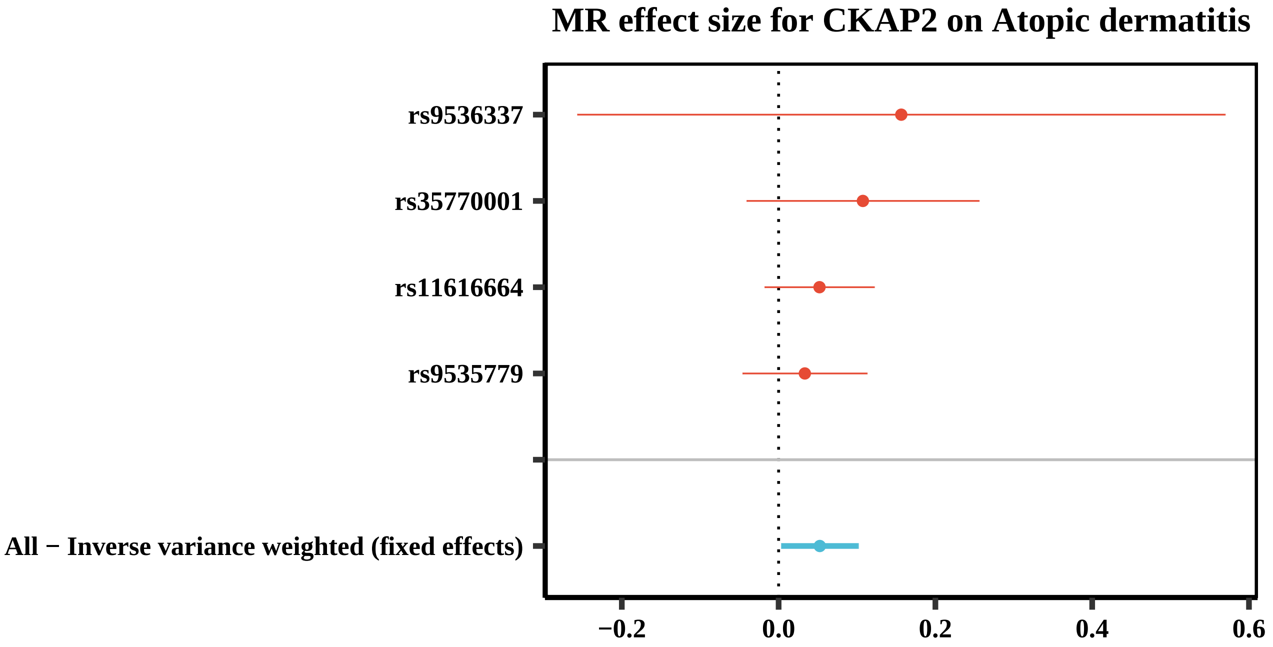

MR effect size for CKAP2 on Atopic dermatitis
rs9536337
rs35770001
rs11616664
rs9535779
All − Inverse variance weighted (fixed effects)
−0.2
0.0
0.2
0.4
0.6
